# How microbes “jeopardize” the modern synthesis

**DOI:** 10.1371/journal.pgen.1008166

**Published:** 2019-05-15

**Authors:** Aaron Novick, W. Ford Doolittle

**Affiliations:** 1 Department of Philosophy, Dalhousie University, Halifax, Nova Scotia, Canada; 2 Department of Biochemistry and Molecular Biology, Dalhousie University, Halifax, Nova Scotia, Canada

The title of this series of reviews is, “How Microbes ‘Jeopardize’ the Modern Synthesis.” To understand how the study of microbial evolution is changing evolutionary theory, it is important to understand what evolutionary theory is. One common view is that the modern synthesis furnished a general, unified theory of evolution, and that new findings about microbes show that this theory is false in important ways and must be replaced by a new, equally general and unified theory [1–3]. But there are other (and we think better) ways to think about this, focused not on theoretical conflict but on theoretical integration [4].

One better way is to see evolutionary theory as a toolkit of explanatory resources, which may be variously combined to explain evolutionary phenomena of interest [5–7]). These resources are neither true nor false in themselves. Rather, they are applicable or inapplicable to particular episodes or patterns in evolutionary history. For example, the Stable Ecotype Model describes a possible pattern of speciation. The model itself is neither true nor false; the appropriate question is how well it captures actual cases of speciation [8]. Perhaps if it never applied, it could be declared false, but in biology one should never say “never.”

This toolkit is the core of evolutionary theory. As it develops, biologists make claims about the range and nature of circumstances in which particular resources are applicable—call these “scope claims”. Scope claims are relative to the state of biological science at a given time: what problems are of most concern, what alternative resources are available, and what organisms are most centrally studied. Scope claims are thus ephemeral products of their time, even as the explanatory resources they concern endure [9].

The strongest claims about microbes “jeopardizing” the modern synthesis turn on (a) identifying the synthetic theory with particular scope claims and (b) stripping those scope claims from their initial context of utterance. For instance, the prevalence of lateral gene transfer (LGT) among microbes might be seen as a challenge to the gradualism of the synthesis. But let us place that gradualism in its historical context. First, the synthesis primarily studied sexually reproducing populations, and often explicitly excluded microbes [10–11]. Second, debates over gradualism in this period primarily concerned the adequacy of gradualistic theories of speciation [12–14]. LGT is not primarily a speciation mechanism; indeed, it rather complicates the very idea of bacterial species [15–18]. Debates about the role of LGT in evolution are by and large not part of the same debate over gradualism that was so central to the synthesis.

The blame for such confusions does not lie entirely with the critics of the synthesis. Proponents have also given undue importance to their scope claims, treating the synthesis as having furnished a general theory of evolution. But there is good reason to think there never was and never can be such a theory, unless it is something so general as to be uninformative, such as: the current diversity and adaptedness of organisms is best understood as the result of genetic and ecological processes (which we for the most part understand) operating over the last four billion years, or so. Evolutionary processes are constrained by the materials (organisms and their variational and populational properties) on which they operate, and these materials are themselves evolved. Evolutionary processes thus produce diverse types of life that are themselves subject to diverse evolutionary processes. Holding out hope for a general theory that applies to all forms of life fails to take seriously one of the main lessons of evolutionary theory, what Beatty calls the “evolutionary contingency thesis” [19].

In light of this, it’s more informative to focus on explanatory resources. When we do so, we can understand models of evolution that incorporate LGT as expanding our toolkit. The study of LGT in microbes does show limitations to the gradualism of the synthesis: the toolkit of the synthesis, while not useless for understanding microbial evolution, is nonetheless not sufficient. As the toolkit is supplemented with new models based on knowledge of previously undiscovered processes, the value of these older resources is clarified without being denied: they are not jeopardized, but their limits are better understood.

Each of the reviews in this series participates, in its own way, in this twofold process of expansion (of our explanatory toolkit) and clarification/limitation (of the scope of existing tools). They show how studying microbes has led to new models of reticulate evolution [20], new models and concepts of speciation [8], new ways of conceiving the ‘genome’ [21], and new ways of understanding mutation [22] and adaptive evolution [23].

In clarifying the scope of existing tools, the study of microbial evolution does more than merely reveal that the resources of the synthesis were limited to macrobes. It also (a) shows how such resources can be extended, with appropriate modifications, to microbes and (b) can even shift our understanding of macrobial evolution. The review on speciation by Shapiro et al. is a good example of both points [8]. First, Shapiro et al. show how ecological approaches to understanding species differences in macrobes [24] can be modified to apply to microbes [25–26]. Second, in light of this, they show how one can place models of macrobial speciation within a broad conceptual framework that incorporates insights from the study of microbial speciation [27]. Clarification of the value of existing resources goes beyond limiting their scope: it also involves making connections between old and new resources.

Scientists today can no more escape their own context than could past scientists. The authors in this series, in addition to presenting novel explanatory resources that have emerged from the study of microbes, inevitably make scope claims of their own—claims that are subject to the limitations of current knowledge. We feel confident in predicting that, in another sixty to eighty years, the explanatory resources presented in these reviews will persist, even as, with new discoveries and new expansions of our toolkit, the biologists of the future will be writing their own reviews about how the evolutionary theory of the early 21^st^ century—which they will identify with the scope claims of today—is being “jeopardized.”
